# The Dynamic Interplay between Appraisal and Core Affect in Daily Life

**DOI:** 10.3389/fpsyg.2012.00380

**Published:** 2012-10-08

**Authors:** Peter Kuppens, Dominique Champagne, Francis Tuerlinckx

**Affiliations:** ^1^Faculty of Psychology and Educational Sciences, KU Leuven-University of LeuvenLeuven, Belgium; ^2^School of Psychological Sciences, University of MelbourneMelbourne, VIC, Australia

**Keywords:** cognition-emotion, core affect, appraisal, experience sampling, daily life

## Abstract

Appraisals and core affect are both considered central to the experience of emotion. In this study we examine the bidirectional relationships between these two components of emotional experience by examining how core affect changes following how people appraise events and how appraisals in turn change following how they feel in daily life. In an experience sampling study, participants recorded their core affect and appraisals of ongoing events; data were analyzed using cross-lagged multilevel modeling. Valence-appraisal relationships were found to be characterized by congruency: the same appraisals that were associated with a change in pleasure-displeasure (motivational congruency, other-agency, coping potential, and future expectancy), changed themselves as a function of pleasure-displeasure. In turn, mainly secondary appraisals of who is responsible and how one is able to cope with events were associated with changes in arousal, which itself is followed by changes in the future appraised relevance of events. These results integrate core affect and appraisal approaches to emotion by demonstrating the dynamic interplay of how appraisals are followed by changes in core affect which in turn change our basis for judging future events.

## Introduction

The interface between cognition and emotion has held a prime position on the research agenda of psychological scientists for decades. If anything, it is clear that there is agreement about two basic facts: that cognition affects emotion, and that emotion affects cognition (Schwartz and Clore, 1983; Ortony et al., 1988; Forgas, 1995, 2000; Boden and Berenbaum, 2010). In the present paper, we address how cognition-emotion relationships play out in both these directions throughout daily life by examining how appraisals are associated with changes in core affect, and how core affect in turn is associated with changes in people’s appraisals.

Appraisals are cognitive evaluations of events that are considered to be the proximal psychological determinants of emotional experience, with different combinations of appraisals corresponding to different emotions (Ellsworth and Scherer, 2003). Several proposals exist in the literature about what constitutes the most important appraisal dimensions, yet they show large overlap and all include appraisals of motivational relevance (is it important?), motivational congruence (is it advantageous or disadvantageous?), agency (is it caused by others or myself?), problem and emotion focused coping potential (can I cope with the situation and with my emotions?), and future expectancy (is the expected outcome desired or not?; e.g., Roseman et al., 1990; Smith and Lazarus, 1993; Scherer, 2009).

Core affect refers to an integral blend of feelings of valence (ranging from pleasure to displeasure) and arousal (ranging from active to passive), which are considered to reflect the most basic dimensions characterizing emotional feelings (Russell, 2003). Core affect is very well suited to examine feelings in daily life. While emotional episodes such as anger, joy, or sadness are relatively infrequent (Zelenski and Larsen, 2000), core affect is continually present and changing across time, and accessible to self-report (Russell, 2003; Kuppens et al., 2007, 2010).

Appraisal and core affect represent two major approaches to understand emotional experience in contemporary emotion research (Scherer et al., 2001; Russell, 2003; Barrett et al., 2007). Yet, the conceptualization of everyday feeling states in terms of core affect and appraisal have largely lived side by side. There is a wealth of research that has addressed how appraisals are related to distinct emotional labels such as fear, anger, sadness, happiness, etc. (see, Scherer et al., 2001) and various biological or more distal determinants of core affect have been identified (Russell, 2003). However, almost no research has examined directly how appraisals impact core affect and vice versa.

### Appraisal influences on core affect

Despite the important role core affect is known to play in many domains of psychological functioning (such as perception, memory, decision making, etc.), there has been relatively little research on what impacts changes in core affect from moment to moment (Russell, 2009).

We argue that one prime candidate for processes that have the ability to change core affect is appraisal. According to appraisal theories of emotions, people continuously evaluate their circumstances in terms of their implication for their own well-being, goals, and concerns. One’s appraisal of the situation as important, advantageous, or disadvantageous, caused by self or another, easy or difficult to cope with, etc. is considered to be the proximal psychological factor in determining the experienced quality of feeling and emotion (Ellsworth and Scherer, 2003).

That appraisals influence core affect (and vice versa) seems obvious and is often acknowledged (Russell, 2003; Barrett et al., 2007; Boden and Berenbaum, 2010). If core affect reflects the fundamental qualities of experienced emotions, moods, and affect (Russell, 2003) and if appraisals represent the proximal determinants of emotional experience (Ellsworth and Scherer, 2003), appraisals should play a primary role in shaping core affect. Moreover, in a way, both the core affect and appraisal perspective represent dimensional views of the emotional realm, with the latter being lower in dimensionality than the former. Even on this basis alone one should expect the two to be strongly related. Yet, despite these obvious connections, very little has been done to bridge these two theoretical viewpoints empirically.

One exception to this is a study by Scherer et al. (2006) who examined how valence and arousal ratings of affective pictures map on appraisal ratings of such pictures. They showed how people’s core affective responses to affectively laden pictures can be predicted by appraisal evaluations of such pictures. However, this study examined responses to standardized pictures, not real-life events. Moreover, different participants provided the core affect and appraisal ratings, precluding any examination of within-person processes in how appraisals may shape core affect or vice versa. As argued by Molenaar and Campbell (2009), within-person processes do not necessarily coincide with between-person processes (or aggregated within-context effects, such as in Scherer et al., 2006), yet it is exactly at the former level that most psychological phenomena play out.

The first aim of the present study is therefore to examine how appraisals impact core affect in daily life. More precisely, we will examine how appraisals made in daily life are associated with consecutive changes in the two dimensions that constitute core affect, pleasure, and arousal. We advance the following hypotheses in this respect.

Motivational relevance is considered to impact the emotional intensity of experienced feelings: the more important an event, the more intense our feelings are in response to it (e.g., Frijda, 1986). On the one hand, it could be argued that arousal reflects emotional intensity, resulting in the prediction that motivational relevance will impact arousal. On the other hand, it has been argued that intensity in core affect terms lies in how extreme one’s feeling is, in terms of its distance from a neutral middle position (Reisenzein, 1994). From that perspective, the appraised importance of an event in itself is not expected to impact the level of arousal (or valence) of one’s feelings.

Motivational congruence, by contrast, is obviously expected to impact the valence dimension of one’s feelings (Scherer et al., 2006): events appraised as advantageous shift core affect toward more pleasant feelings, while events appraised as disadvantageous create more displeasure.

According to appraisal theories, appraisals of agency (whether oneself or another person is responsible) are in themselves not expected to impact pleasure or arousal in a particular direction (e.g., Frijda, 1986; Roseman et al., 1990; Smith and Lazarus, 1993; Scherer, 2009). While in combination with appraisals of advantageousness/disadvantageousness they are considered to be implicated in feelings of anger or pride, shame, and guilt (with corresponding changes in the core affect space), we do not have strong *a priori* expectations how blaming oneself or someone else for what has happened would in itself impact valence or arousal.

Problem and emotion focused coping potential, on the other hand, are expected to cause feelings to be more pleasant and less unpleasant, based on the large literature of these processes in the coping domain (Lazarus and Folkman, 1984; Folkman and Lazarus, 1985). Low coping potential is indeed mainly implicated in the elicitation of negative emotions, not positive emotions (Smith and Lazarus, 1993). If one feels one can cope with the situation or with the emotions elicited by the situation, one can reasonably assume that this will have a positive effect on how pleasant one is feeling (or a negative one on how unpleasant one is feeling), regardless of the distinct quality of that feeling, and vice versa. In addition, particularly problem focused coping potential may be expected to increase arousal. To the extent that arousal can be regarded as reflecting the level of goal pursuit that is involved in the generation of affect (with high arousal emotions such as anger and happiness involving high levels of goal pursuit and low arousal emotions such as sadness and relaxation involving the abandonment of goal pursuit; Carver and Scheier, 1990), we hypothesize that problem focused coping potential will engage active goal pursuit, and accordingly increase arousal levels (which may, speculatively, reflect the physiological mobilization of needed energy resources).

Finally, we expect that future expectancy will generate more pleasant feelings and less unpleasant feelings, consistent with findings that for instance optimism increases positive mood (Segerstrom et al., 1998). Especially in the model by Smith and Lazarus (1993), in which this appraisal is conceptualized as the certainty that things will turn out how one wants, the link with increase in valence is obvious. In addition, negative future expectancy may generate feelings of stress and anxiety (Smith and Lazarus, 1993), which may translate to higher levels of arousal.

Although some of these predictions about how appraisals impact core affect are obvious, they nevertheless remain largely theoretical and require empirical verification. Not only does a study that addresses these relationship add to our knowledge about what determines core affect, it also complements appraisal theory by moving its empirical basis beyond the domain of modal emotions and applying it to core affect.

In the present paper, we chose to examine the simple effects of appraisals on core affect, and not how combinations, patterns, or interactions of appraisals impact core affect. While the latter is certainly important (e.g., Tong, 2010a), the specific research questions also rise exponentially in number. Therefore, to keep things simple at this stage, we examine how individual appraisals, in themselves and not in combination with others, influence core affect.

### Core affect influences on appraisal

How we feel, in turn, provides a basis for the judgments we make about our world. According to the affect-as-information view, subjectively experienced feelings are used as important pointers for evaluating one’s relationship with the environment (Clore and Huntsinger, 2007). As a result, our current mood is often reflected in our judgments (Boden and Berenbaum, 2010). For instance, studies investigating well-being (Schwartz and Clore, 1983), social justice (van den Bos, 2003), or consumer judgment (Yeung and Wyer, 2004) have shown that people rate their satisfaction with life, the justness of their decisions, and even characteristics of consumer products to be more positive when they feel happy, and more negative when they feel sad.

Lerner and Keltner (2000, 2001), starting from a similar assumption, argued that a person’s emotional state gives rise to the tendency to appraise novel events in ways congruent with the appraisals associated with their emotion. According to this appraisal tendency framework, for instance, anger (which is considered to be associated with high coping potential) leads people to appraise their circumstances as more controllable (hence leading to riskier choices), whereas fear has the opposite effect.

Building on the affect-as-information account and appraisal tendency framework, we can formulate predictions about how core affect might bring about changes in people’s appraisals. First, in line with the idea of mood congruency (Russell, 2003), we expect that a more pleasant affect will lead people to appraise future events as more advantageous, involving more coping potential, and more optimistic future expectancy. Indeed, quite some research has shown that being in a positive mood state makes people more optimistic, to have more confidence in their own coping capabilities (Lyubomirsky et al., 2005), and so on. Further, it has been shown that people who are in a more pleasant core affective state make more positive simulations about the future (Sanna, 1999).

Second, in terms of arousal impacting appraisals, predictions are less clear. Given that arousal is not linearly related to valence (e.g., Reisenzein, 1994), we don’t expect arousal to impact appraisals of motivational congruence. Furthermore, on the one hand, high arousal is associated with active approach emotions such as excitement and anger, which could translate into appraisals of personal agency, high coping potential, and future expectancy. Yet, high arousal is also present in feelings of fear and anxiety, which are associated with the opposite pattern of these appraisals. The only appraisal dimension we expect to be potentially influenced by arousal, is motivational relevance. Arousal, as has been argued, provides an attention-capturing signal (Boden and Berenbaum, 2010). A state of high arousal therefore could be thought to create a state of emotional vigilance in an individual, in which future events are interpreted as more relevant for one’s own concerns. Indeed, both arousal and vigilance are considered to rely on overlapping brain systems with a central role for the amygdala (Davis and Whalen, 2001), which itself is seen to act as the relevance detector of the brain (Sander et al., 2003).

To summarize, in this study we examine how appraising the world in a certain way is associated with changes in core affect, and vice versa. We addressed our research questions in an experience sampling study in which participants reported on their core affect and how they appraised their current circumstances several times a day over a 2-week period. Cross-lagged multilevel analyses were used to determine how appraisals are associated with changes in core affect from one moment to the next, and vice versa.

## Materials and Methods

### Participants

Eighty university students took part in the study. One participant ended the study after one day, resulting in a final sample of 79 participants (50 females, 29 males; mean age = 24 years). Participants were paid 40€ for their participation.

### Materials

#### Repeated assessment of core affect

Core affect was assessed at each sampling moment using a modified version of the Affect Grid, a single-item measure designed to simultaneously assess subjectively felt valence and arousal (Russell et al., 1989). The modified version consisted of a 99 × 99 (instead of 9 × 9) two-dimensional grid, with a neutral middle row and middle column. Unpleasant/pleasant feelings form the horizontal dimension, passive/active arousal the vertical. End- and midpoints are marked with affective labels to facilitate reporting. Participants were instructed to mark the position on the Affect Grid that best corresponded to how they felt at each sampling moment (signaled by a beep). The one-item Affect Grid is ideally suited for repeatedly assessing core affect in the context of experience sampling as it does not overload participants and enables a quick response.

#### Repeated assessment of appraisal dimensions

At each sampling moment, participants were also asked to report how they appraised the current events that determined their feelings at that moment by responding to seven questions using a continuous slider scale that, in all but one case, ranged from 0 (*not at all*) to 100 (*very much*). The seven appraisal items reflected the dimensions proposed in the framework by Smith and Lazarus (1993) and were adapted for use in the context of an experience sampling study. Each item started with the prompt “Think about what is causing your feelings right now,” followed by “to what extent is this important for you?” (motivational relevance), “To what extent is this advantageous or disadvantageous to you?” (motivational congruence, with the response ranging from −50 (*very disadvantageous for me*) to +50 (*very advantageous for me*), “To what extent is someone else responsible for this?” (other-agency), “To what extent are you yourself responsible for this?” (self-agency), “To what extent do you think you can change something about this situation?” (problem focused coping potential), “To what extent do you think you can emotionally cope with this?” (emotion focused coping potential), and “to what extent do you think events will turn out the way you want?” (future expectancy). These items were presented in a randomized order at each beep.

### Procedure

In a first session, each participant received a Tungsten E2 palmtop computer along with instructions for its use in general as well as for responding to the questions at each beep, including elaborated instructions for the Affect Grid (see, Russell et al., 1989). Each palmtop was programmed to beep 10 times a day for 14 consecutive days during the participant’s waking hours using the Experience Sampling Program (Barrett and Barrett, 2001). The beeps were programmed according to a stratified random interval scheme: participant’s waking hours were divided into 10 equal intervals and one beep was programmed randomly in each interval. At each beep, the palmtop presented a series of questions: first the Affect Grid item, followed by the appraisal items of which the order was randomized at each beep. For the next 2 weeks, participants carried the palmtop during their normal daily activities and responded to the questions when signaled. Compliance was good: overall, participants responded to 82% of the programmed beeps (response frequency ranged between 55 and 99% for individual participants). After 2 weeks, participants attended a second session in which they were debriefed and paid for participation.

## Results

Table 1 lists descriptive statistics for the core affect and appraisal dimensions and their intercorrelations (calculated using multilevel modeling, see Nezlek, 2012). In line with the discussed literature, the correlations show that pleasure is mainly associated with motivational congruence, coping potential, and future expectancy, while arousal is less strongly concurrently associated with appraisals. Pleasure was in addition found to be positively associated with self-agency, and negatively (albeit relatively weakly) with other-agency. While these appraisals are generally tied to emotions in combination with other appraisals, these results suggest that self-vs. other-agency has a pleasant vs. unpleasant connotation to it as well. Also the appraisals themselves show several meaningful interrelations.

**Table 1:** **Descriptive statistics and multilevel correlations of core affect and appraisal categories**.

	N	Mean	SD	Pleas.^(A)^	Act.^(B)^	M.R.^(C)^	M.C.^(D)^	O.A.^(E)^	S.A.^(F)^	P.F.C.^(G)^	E.F.C.^(H)^	F.E.^(I)^
Pleas.^(A)^	8380	59.02	19.45	–	–	–	–	–	–	–	–	–
Act.^(B)^	8380	44.54	22.78	−0.35**	–	–	–	–	–	–	–	–
M.R.^(C)^	9381	63.60	23.09	0.20**	−0.15**	–	–	–	–	–	–	–
M.C.^(D)^	9380	56.46	21.31	0.49**	−0.19**	0.32**	–	–	–	–	–	–
O.A.^(E)^	9379	44.66	28.91	−0.17**	−0.14**	0.17**	0.22**	–	–	–	–	–
S.A.^(F)^	9376	59.95	25.20	0.26**	−0.12**	0.20**	0.34**	0.52**	–	–	–	–
P.F.C.^(G)^	9380	57.14	24.38	0.26**	−0.12**	0.21**	0.36**	0.20**	0.38**	–	–	–
E.F.C.^(H)^	9377	72.88	20.14	0.37**	−0.14**	−0.22**	0.48**	−0.21**	0.28**	0.30**	–	–
F.E.^(I)^	9376	61.12	21.72	0.38**	−0.15**	0.20**	0.52**	−0.22**	−0.35**	0.39**	0.46**	–

***p < 0.001 level. ^(A)^Pleasure, ^(B)^Activation, ^(C)^Motivational relevance, ^(D)^Motivational congruency, ^(E)^Other-agency, ^(F)^Self-agency, ^(G)^Problem focused coping potential, ^(H)^Emotion focused coping potential, ^(I)^Future expectancy.

Our main research question concerned how current appraisals are associated with subsequent changes in core affect and vice versa. To answer this question, we used multilevel (to take into account the nested data structure and resulting dependencies) autocorrelation-crosscorrelation regression models to examine how each of the appraisals at one point in time is associated with a subsequent change in valence or arousal, and vice versa. For instance, valence at time t was predicted by valence at time *t*−1 and an appraisal at time *t*−1 at level 1 of the models:

$$Valence_{t,j}=\beta_{0j}+\beta_{0j}Valence_{t-1,j}+\beta_{2j}Appraisal_{t-1,j}+\varepsilon_{t,j}$$

In such a model, the effect of the appraisal at time *t*−1 reflects how this appraisal is associated with a change in valence from *t*−1 to *t*. For instance, how feeling responsible on one moment is followed by an increase in feeling pleasant. Although this analytical approach does not prove strict causality (because of possible third variables; see more in the limitations section), it is the closest one can come to examining directional and causal relationships on the basis of time series data (Granger, 1969; Gottman, 1990)^1^. In all analyses, predictor variables were group-mean centered (see, Enders and Tofighi, 2007), intercept and slope coefficients were allowed to vary across persons, and previous day lagged values were set as missing to avoid between-day effects.

### Appraisals predicting core affect

**Figure 1:**
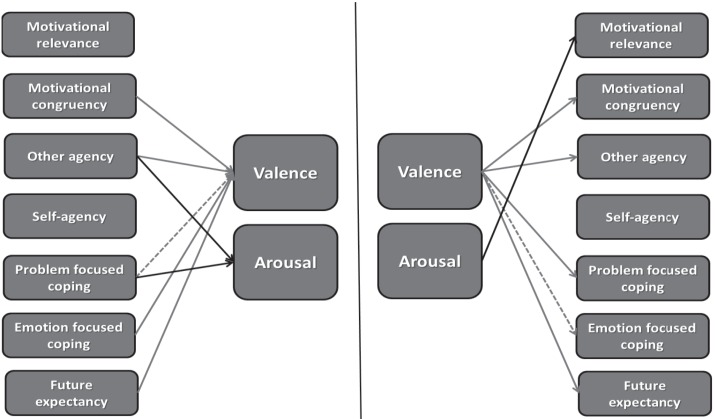
**Graphical summary of directional appraisal-core affect (left panel) and core affect-appraisal relationships (right panel) based on separate analyses each involving one core affect dimension and one appraisal**. Full lines represent significant relationships with *p* < 0.05, dotted lines represent marginally significant relationships with *p* < 0.10.

First, we estimated multilevel models in which one core affect dimension was predicted by one appraisal variable each time, the results of which can be found in Table 2 (and graphically summarized in the left panel of Figure 1). They show that some but not all appraisals are associated with changes in core affect. As expected, appraised advantageousness, problem (marginally significant), and emotion focused coping potential, and future expectancy were associated with increases in pleasant valence, meaning that appraising events in these ways was followed by participants feeling more pleasant and less unpleasant from the moment they reported this appraisal till the moment of the next assessment. Also consistent with predictions, we found that arousal increased after appraising events as high in problem focused coping potential, though not after appraising events as more motivationally relevance. Finally, although not specifically predicted, we also found that appraising events as being caused by someone else was associated with increased valence and decreased arousal.

**Table 2:** **Results from cross-lagged multilevel regression analyses predicting change in valence or arousal on the basis of lagged appraisal**.

Appraisal (predictor)	Valence (criterion)				Arousal (criterion)			
	B	SE	SD^a^	Pseudo-*R*^2^^b^	B	SE	SD^a^	Pseudo-*R*^2^^b^
Motivational relevance	0.012	0.012	0.056^†^	0.00252	−0.001	0.012	0.026	−0.00031
Motivational congruence	0.075***	0.015	0.080***	0.00954	−0.009	0.015	0.065*	0.00276
Other-agency	0.019*	0.009	0.044*	0.00295	−0.022*	0.010	0.046	0.00314
Self-agency	0.014	0.010	0.039	0.00099	0.012	0.012	0.059*	0.00307
Problem focused coping	0.021^†^	0.011	0.043*	0.00127	0.027*	0.011	0.026	0.0062
Emotion focused coping	0.045**	0.014	0.060*	0.00305	0.003	0.014	0.034	−0.00045
Future expectancy	0.065***	0.013	0.64^†^	0.0052	0.003	0.016	0.078*	0.00366

*^a^Standard deviation of random slope of lagged appraisal predicting change in core affect dimension*.

*^b^Pseudo *R*-squared was calculated following Singer and Willet (2003)*.

***p < 0.001, **p < 0.01, *p < 0.05, ^†^*p* < 0.06.

As an aside, in several cases we also observed large and significant variance across persons in the slopes reflecting the effect of the appraisals on core affect (see Table 2), reflecting that people differ in how strongly the appraisals shape core affect.

### Core affect predicting appraisal

Next, we estimated models to reveal how valence and arousal themselves are associated with changes in the individual appraisals to the next point in time. The results of the analyses are reported in Table 3 (and summarized in the right panel of Figure 1). They show that core affect is associated with changes in some, but not all appraisal categories. Consistent with our predictions, we found that the more people were in a pleasant core affective state, the more likely they would appraise future events more favorably in terms of advantageousness and their problem and emotion (marginally significant) coping potential. They also tended to hold more positive future expectations about these events. In terms of arousal predicting appraisals, we found that the more aroused people were, the more they would consider future events as important to them. Finally, although not predicted, we found that appraisals of other-agency (other accountability) increased when participants felt more pleasant. Again, we observed large individual differences in these effects.

**Table 3:** **Results from cross-lagged multilevel regression analyses predicting change in appraisal based on lagged valence or arousal**.

Appraisal (criterion)	Valence (predictor)				Arousal (predictor)			
	B	SE	SD^a^	Pseudo-*R*^2^^b^	B	SE	SD^a^	Pseudo-*R*^2^^b^
Motivational relevance	0.002	0.014	0.048	0.00159	0.029*	0.012	0.055*	0.00478
Motivational congruence	0.049**	0.018	0.100***	0.00838	0.014	0.012	0.057*	0.00447
Other-agency	0.089***	0.021	0.113***	0.0088	−0.004	0.015	0.067**	0.00299
Self-agency	0.000	0.020	0.113***	0.00676	0.014	0.014	0.062*	0.00486
Problem focused coping	0.054**	0.017	0.081**	0.00643	0.016	0.012	0.040	0.00179
Emotion focused coping	0.030^†^	0.016	0.091***	0.00854	0.009	0.009	0.032*	0.00155
Future expectancy	0.036*	0.016	0.081**	0.00664	0.011	0.012	0.066***	0.00626

*^a^Standard deviation of random slope of lagged core affect dimension predicting change in appraisal*.

*^b^Pseudo *R*-squared was calculated following Singer and Willet (2003)*.

***p < 0.001, **p < 0.01, *p < 0.05, ^†^*p* < 0.06.

## Discussion

Core affect and appraisal play the lead roles in two of the most dominant accounts of what determines or constitutes emotional experience. In the present study, we examined how these prominent cognitive and affective components of emotional experience dynamically relate to each other in daily life.

The findings revealed insightful patterns of the continuous interplay between core affect and appraisal. Consistent with appraisal theory predictions, participants felt more pleasant after appraising events as high in motivational congruency, problem and emotion focused potential, and optimistic future expectancy, and vice versa. Feeling pleasant, in turn, was itself also associated with appraising future events as more motivationally congruent, easier to cope with, and being more optimistic about their outcome. In addition, it was found that appraising events as caused by someone else was also associated with feeling pleasant, and vice versa. This was an unexpected finding, but resonates with research that links positive mood to increased fundamental attribution error (Forgas, 1998).

In sum, valence-appraisal dynamics are characterized by a principle of congruency: the ways feeling pleasant changes as a function of appraisals is mirrored by how these appraisals change as a function of feeling pleasant. On the one hand, this congruency may reflect a self-reinforcing feedback cycle that may help to maintain a positive, optimistic mood. On the other hand, it may also assist in creating a more dysfunctional cycle of negative moods and negative cognitions as observed in for instance depression (Joormann, 2010).

A different pattern emerged for arousal. Arousal increased when participants perceived events as controllable and decreased when events were perceived to be caused by others. We did not find support for the notion that motivational relevance increased arousal. Together, these results suggest that arousal may primarily be a function of feeling in control of the outcome of the situation, something which is arguably lower when it is caused by someone else. It can be conjectured that the increased arousal reflects the activation needed to influence the outcome of a situation when deemed feasible.

Arousal was not characterized by congruency in terms of its relationships with appraisals, however, in that levels of arousals were not found to be associated with changes in these same appraisals. Instead, higher levels of arousal were associated with increasingly appraising events as more personally relevant. This finding suggests that higher arousal may create a sort of vigilant state that, as a default, tends to interpret future events as more personally relevant, possibly to facilitate a quick and efficient response. To the extent that arousal reflects active goal pursuit, the observed pattern of results suggests that arousal is increased when the situation leaves opportunity to impact on it (e.g., when not caused by others and controllable), while it itself creates an appraisal mindset that heightens the importance of future events in relation to one’s goals to act upon them as soon as necessary. Arousal, therefore, may play a critical role in implicating an individual in its environment.

On a more abstract level, the present findings illustrate how changes in affect and judgments about the world follow from each other in an ongoing manner throughout people’s daily lives. Among other things, this interplay between appraisals and core affect may contribute to the sense of continuity that we experience in our emotional lives. While specific emotions may be elicited by separate events, we nevertheless experience a remarkable continuity in the feelings we experience on a daily basis. The fact that appraisals and core affect become intertwined across time may play a significant role to establish this.

While not of primary interest to our research question, we found substantial individual differences in the ways appraisals and core affect mutually follow each other across time. This resonates with other findings showing large and meaningful individual differences in the relationships between appraisals and discrete emotional experiences (e.g., Kuppens et al., 2008; Tong, 2010b; see Kuppens and Tong, 2010, for an overview). In future research, it will be important to pinpoint the nature of these individual differences in terms of how they relate to personality and emotion regulation processes.

While the current study is characterized by a number of strengths, such as high ecological validity and the use of appropriate statistical modeling, it also suffers from the weakness that our data solely rely on self-report. Yet, there is currently no valid alternative to know how people feel than to ask them. While self-reports of appraisal and core affect dimensions may be contaminated by response biases, we tried to limit the impact of such biases as much as possible by only asking participants questions on what they were experiencing at the current moment of assessment. Another limitation is that experience sampling methods do not allow controlling for or holding constant the types of events people encounter in daily life. As a result, it is not possible to rule out the possibility that found relationships actually reflect how events or the anticipation of them may lie at the base of how core affect changes as a function of appraisals (e.g., future expectancy is high now because I anticipate an event that will increase my pleasure from now till the next moment) or how appraisals change as a function of feelings (e.g., my arousal now is high because I expect a personally relevant event to occur). Experimental research that allows controlling situational variance is needed to establish full causality. Finally, the appraisal and core affect changes were observed over the course of minutes and hours (the average time lag between observations being approx. 66 min). Clearly, core affect and appraisal impact each other on a much shorter time scale, but these rapid changes are difficult to detect using daily life methodology. The fact that we nevertheless found meaningful patterns of mutual appraisal-core affect relationship in this study attest that the observed changes are relatively lasting.

To conclude, the findings from this study underscore that appraisals and core affect are not independent properties of emotional experience, but are intricately related in a dynamic interplay that is characterized by congruency between appraisals and valence and that suggests a central role in acting and responding on the environment for arousal. How we appraise changes following how we feel, which in turn changes following how we perceive future events. Emotions or moods should therefore not be understood as static phases, but as dynamic phenomena of which the components continuously change and follow each other across time (Kuppens et al., 2009; Boden and Berenbaum, 2010).

## Conflict of Interest Statement

The authors declare that the research was conducted in the absence of any commercial or financial relationships that could be construed as a potential conflict of interest.

## Footnotes

^1^This type of statistical models is also known as VAR models (Hamilton, 1994; Lütkepohl, 2005) or simple causal models (Granger, 1969). VAR models are closely related to so-called Structural VAR (SVAR) or instantaneous causality models (Granger, 1969; Lütkepohl, 2005), which additionally include the predictor variable at time *t* into the model. In the present context, it was decided to work with a VAR modeling approach because of a combination of methodological and theoretical considerations. These involved the fact that it is impossible to formulate directional conclusions based on simultaneous relationships in this type of data where appraisals and core affect are theoretically assumed to mutually influence each other, possible problems of multicollinearity and problematic interpretation of results when including predictor variables at time *t*, and a violation of assumptions of uncorrelated error terms within an SVAR approach.
